# scTEA-db: a comprehensive database of novel terminal exon isoforms identified from human single cell transcriptomes

**DOI:** 10.1093/nar/gkad878

**Published:** 2023-10-18

**Authors:** Miguel Barquin, Ian U Kouzel, Beat Ehrmann, Michael Basler, Andreas J Gruber

**Affiliations:** Department of Biology, University of Konstanz, 78464 Konstanz, Germany; Department of Biology, University of Konstanz, 78464 Konstanz, Germany; Department of Biology, University of Konstanz, 78464 Konstanz, Germany; Department of Biology, University of Konstanz, 78464 Konstanz, Germany; Biotechnology Institute Thurgau (BITg) at the University of Konstanz, 8280, Kreuzlingen, Switzerland; Department of Biology, University of Konstanz, 78464 Konstanz, Germany

## Abstract

The usage of alternative terminal exons results in messenger RNA (mRNA) isoforms that differ in their 3′ untranslated regions (3′ UTRs) and often also in their protein-coding sequences. Alternative 3′ UTRs contain different sets of *cis*-regulatory elements known to regulate mRNA stability, translation and localization, all of which are vital to cell identity and function. In previous work, we revealed that ∼25 percent of the experimentally observed RNA 3′ ends are located within regions currently annotated as intronic, indicating that many 3′ end isoforms remain to be uncovered. Also, the inclusion of not yet annotated terminal exons is more tissue specific compared to the already annotated ones. Here, we present the single cell-based Terminal Exon Annotation database (scTEA-db, www.scTEA-db.org) that provides the community with 12 063 so far not yet annotated terminal exons and associated transcript isoforms identified by analysing 53 069 publicly available single cell transcriptomes. Our scTEA-db web portal offers an array of features to find and explore novel terminal exons belonging to 5538 human genes, 110 of which are known cancer drivers. In summary, scTEA-db provides the foundation for studying the biological role of large numbers of so far not annotated terminal exon isoforms in cell identity and function.

## Graphical Abstract

**Graphical Abstract:**
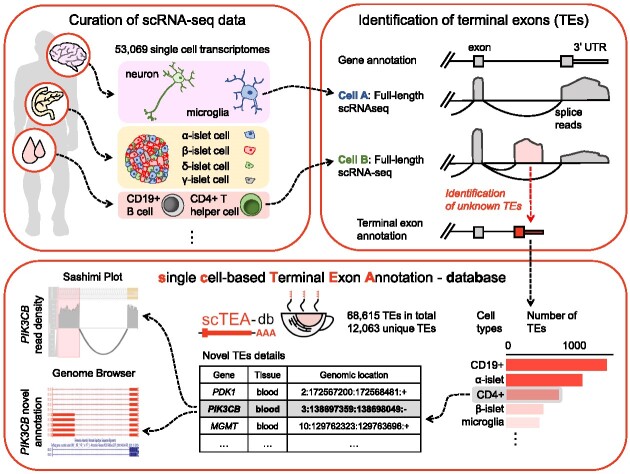

## Introduction

Most of the transcript isoform expression variation across human tissues is caused by the use of alternative promoters and alternative transcript 3′ ends (1). The latter are generated by endonucleolytic cleavage and polyadenylation of the nascent RNA, which is mediated by a large molecular machinery, the so-called 3′ end processing complex. The complex recognizes specific sequence motifs located in the vicinity of the 3′ end processing sites, also called polyadenylation [poly(A)] sites (2). The most prominent RNA 3′ end processing signal is the hexameric consensus motif ‘AAUAAA’ (3), termed canonical poly(A) signal, which is positioned ∼21 nucleotides upstream of poly(A) sites.

The vast majority of human genes have multiple poly(A) sites (4) and the alternative cleavage and polyadenylation (APA) of these sites gives rise to alternative terminal exons that can encode for alternative coding sequences and/or 3′ untranslated regions (3′ UTRs). The latter harbour *cis*-regulatory elements, such as microRNA and RNA binding protein (RBP) binding sites, known to impact the stability, translation and localization of the transcripts (2) and even the localization of the encoded proteins (5).

In humans, the repertoire of 3′ UTRs varies substantially across tissues. Whereas in ovary, testis and embryonic stem cells, 3′ UTRs are short, the longest 3′ UTRs are found in neurons (6). Importantly, APA is dynamically regulated (7) and was reported to play important roles in cell identity and function. For example, the switching of alternative terminal exons is key to neuronal differentiation, where hundreds of genes switch towards using alternative terminal exons whose 3′ UTRs contain binding sites for the muscleblind-like protein 1 (MBNL1) and MBNL2 RBPs. Strikingly, the MBNL1/2 RBP binding sites mediate the transport of the alternative transcript isoforms from the cell soma to the neural projections (8).

Despite the important role that accurate poly(A) site use plays in cell identity and healthy cell function [reviewed in (2)], large numbers of 3′ end transcript isoforms appear to be unknown to date and thus remain to be identified and annotated. Various research groups have developed RNA sequencing protocols, termed 3′ end sequencing, that are specifically designed to capture the 3′ end of transcripts (2). In previous work, we have performed a comprehensive analysis of 3′ end sequencing datasets and found that 24.8% of the detected transcript 3′ ends are located within regions currently annotated as intronic (9). To enable the identification and annotation of transcript isoforms that end at intronic loci, we have previously developed the Terminal Exon Characterization tool (TECtool). Using TECtool to study full-length single cell RNA sequencing (scRNA-seq) data of 201 T cells, we unveiled that there exists an abundance of unknown 3′ end transcript isoforms (10).

Within this study, we have followed up our initial screening of 201 T cells at much larger scale in order to identify and annotate currently unknown terminal exons and corresponding 3′ end isoforms at single cell resolution, covering 101 human cell types and 23 tissues (Table 1). Our single cell-based Terminal Exon Annotation database (scTEA-db, www.scTEA-db.org) provides an abundance of so far unknown terminal exons (68 615 cases, 12 063 of which are unique). scTEA-db not only offers a vastly extended 3′ end isoform annotation, but also implements an array of functions that allow users to find and investigate terminal exons of interest, thereby providing the community with a tool required for studying the biology of large numbers of so far unexplored alternative terminal exon transcript isoforms.

**Table 1.** scTEA-db data summary

**Tissues**	**Novel TEs**	**Cells**	**Cell types**	**Datasets**
Adrenal gland	5	15	1	1
Blastocyst	2893	9882	3	4
Blood	2800	12 973	14	14
Bone	1502	1143	28	4
Bone marrow	1500	3107	27	3
Central nervous system	1649	1397	7	3
Caudal	1279	661	11	1
Colon	74	528	1	1
Decidua	1413	1255	5	1
Early blastomere	2307	89	3	1
Endoderm	47	906	1	1
Fallopian tube	671	1857	1	1
Ovary	156	120	3	2
Testis	1	11	1	1
Lymph node	446	365	3	1
Heart	327	303	1	1
Liver	556	888	2	1
Pancreas	5212	9670	14	5
Preimp. stem cells	202	34	1	1
Rostral	867	336	8	1
Skin	1240	6352	7	7
Tonsil	384	987	5	2
Yolk sac	632	190	5	1

For each tissue (sorted alphabetically), the number of novel terminal exons (TEs), the number of considered cells, the number of cell types and the number of analysed datasets are shown.

## Materials and methods

**Figure 1.**
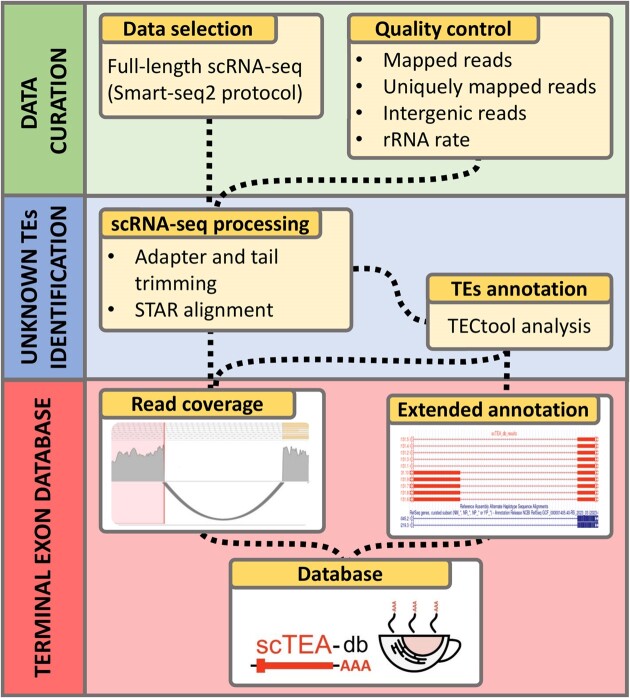
Schematic representation of the scTEA-db data curation, processing and integration. After scRNA-seq data selection and quality control (top panel in green), the sequencing reads of samples with sufficient quality are processed and aligned to the human reference genome using the STAR aligner (16). Subsequently, unknown terminal exons (TEs) and associated isoforms are annotated using TECtool (10) (middle panel in blue). scTEA-db enables users to interactively filter and explore the terminal exon data across cell types and tissues (bottom panel in red).

The scTEA-db data selection, analysis and integration workflow (Figure 1) encompassed the steps outlined below.

### Dataset selection and downloading

Dataset selection exclusively focused on full transcript length-based sequencing technology, in particular scRNA-seq libraries created with the Smart-seq2 protocol (11), enabling the identification and annotation of terminal exons and isoforms using TECtool (10).

To obtain datasets suitable for our analysis, publicly available Smart-seq2 datasets were collected (Supplementary Tables S1 and S2) from the NCBI-SRA (12) and the EMBL-EBI (13) databases based on a set of selection criteria (see Supplementary Materials). The download of the data was carried out using the SRAtoolkit 3.0.3 (12) with default parameters.

### Data processing

The data analysis workflows were implemented using the Snakemake workflow management system (14). The analysis was split into two major workflows: first, the data quality control part (Supplementary Figure S1), and second, the identification of novel terminal exons and isoforms (Supplementary Figure S2).

In the first part, data quality control was performed by aligning the sequencing reads to the human genome version GRCh38.102 (15) and Ensembl gene annotation version GRCh38.102 (15), which was carried out using the STAR aligner version 2.7.1a (16). Then, the following criteria were used to filter out low-quality scRNA-seq libraries: a minimum sequencing read mapping rate of 0.50 and a minimum unique mapping rate of 0.50 as reported by the STAR aligner (16). Further, a maximum intergenic mapping rate of 0.50 and a maximum ribosomal RNA rate of 0.50 were tolerated, both of which were obtained using the RSeQC tool (17).

In the second part, the identification and annotation of novel terminal exons and associated isoforms was performed using TECtool (10). As TECtool runs on single-end read alignments, a Snakemake pipeline was used that first utilizes the STAR aligner to map the sequencing reads in single-end mode; i.e. the alignment was conducted for each sequencing read in pair (fastq1 and fastq2 files) separately. Then, TECtool was run on each BAM file using poly(A) site annotations from the PolyASite 2.0 atlas (18) and the same Ensembl gene annotation and genome versions as utilized for the first part (see above). Finally, Sashimi plots (19) were created for each terminal exon identified and annotated by TECtool (10).

### Data integration and curation

The StringTie tool (20) was used to merge and unify the terminal exon isoforms identified from individual single cells (see Supplementary Materials). The nucleotide frequencies around the 3′ ends of novel terminal exons were comparable to those observed around already annotated terminal exons (Supplementary Figure S3). Finally, the cell type and tissue nomenclature was unified by using the manually curated mapping reported in Supplementary Table S3.

### scTEA-db web application

The scTEA-db web portal was developed using R Shiny (21) and tidyverse (22) packages. The human body map that visualizes the number of terminal exons identified in each tissue was implemented using the ‘gganatogram’ R package (23). The scTEA-db Shiny application’s layout design was further customized making use of hypertext markup language and cascading style sheets.

## Results

**Figure 2.**
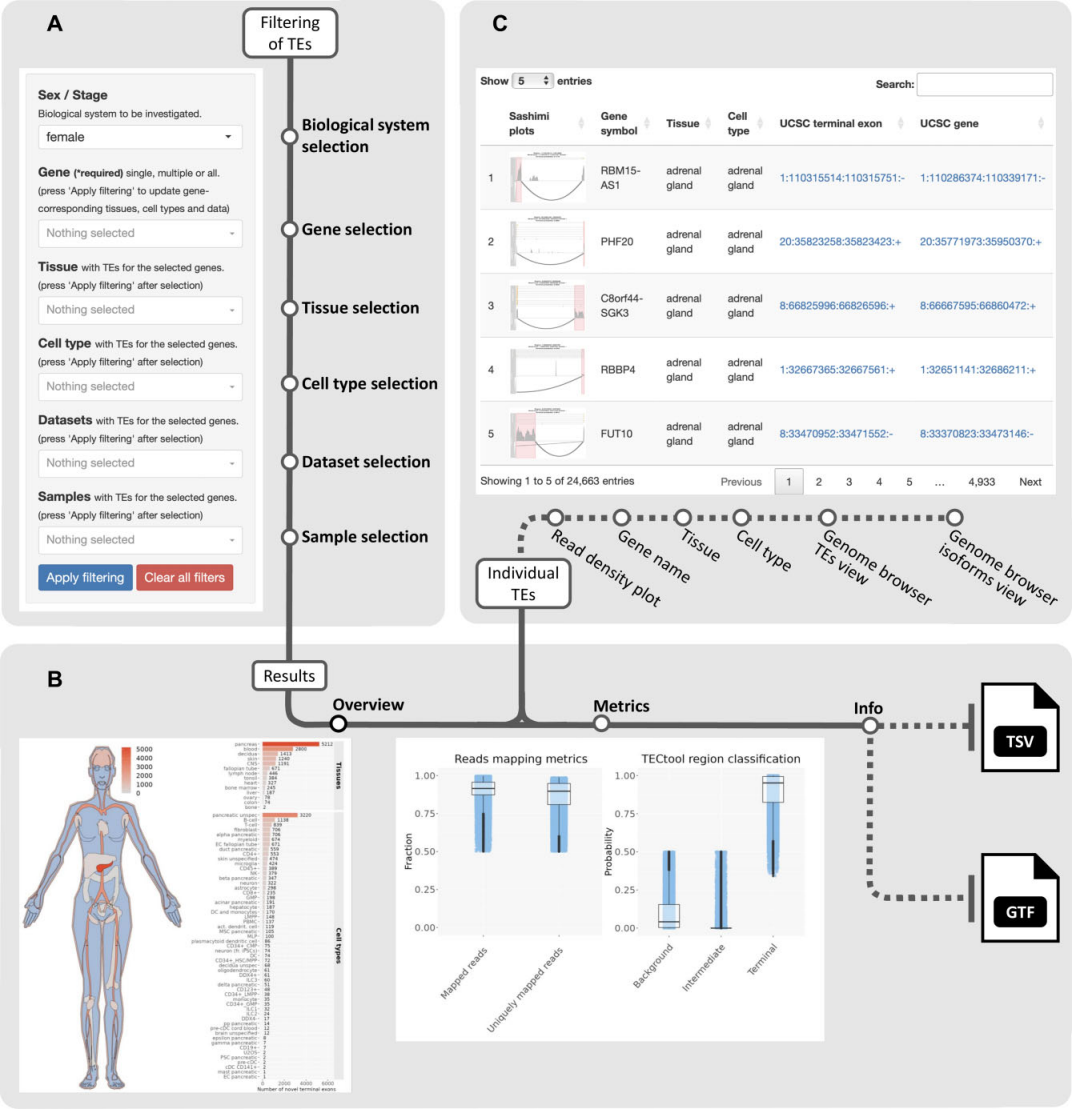
Finding and exploring terminal exons (TEs) using scTEA-db. (**A**) Users can filter the terminal exons by multiple features, such as gene symbol, cell type or tissue. (**B**) The ‘Overview’ and ‘Metrics’ panels provide details about the selected terminal exons. The ‘Info’ panel provides general information about scTEA-db and data bulk download options in tab-separated values (TSV) format and gene transfer format (GTF). (**C**) Details about the selected terminal exons at single cell resolution, including links to the UCSC Genome Browser (24).

scTEA-db is available at www.scTEA-db.org and supports secure communication and data transfer between the web portal and the user. scTEA-db can be accessed via desktop computers and mobile devices, whereas our recommendation is to use it in combination with a decently large electronic visual display to allow for an optimal experience. Below, we provide a roadmap describing the main features and functionalities offered by the scTEA-db web portal (Figure 2).

### Finding terminal exons of interest

To enable users to find terminal exons of their interest, the scTEA-db web portal selection menu (Figure 2A) provides the following filtering options:

*‘Sex/Stage’*: Enables users to select a biological system of interest. Currently possible options are male, female, foetal or any. The latter enables researchers to investigate all terminal exons available within scTEA-db, i.e. independent of their detection within a specific biological system of interest.

*‘Gene’ (required field)*: Allows to search for terminal exons that belong to specific genes. The user is free to search for an individual gene of interest, a set of genes or all genes (option ‘Select All’). When the latter is chosen, all genes for which terminal exons have been identified within the selected biological system of interest will be considered. Please note that the downstream selectors ‘Tissue’, ‘Cell type’, ‘Dataset’ and ‘Samples’ will be automatically updated to show only values available for the selected gene or gene set.

*‘Tissue’, ‘Cell type’, ‘Datasets’ and ‘Samples’ selectors*: The user can further limit the search to specific tissues, cell types, and even datasets and samples. As done for the ‘Gene’ selector, also here all downstream selectors will automatically be filtered according to the user selection; e.g. in case of selecting a specific tissue, only those cell type(s) will remain that are part of this tissue.

Once all selectors are filled, the corresponding filtering is performed upon pressing the ‘Apply filtering’ button. To clear all selectors and start a new search, the user simply needs to make use of the ‘Clear all filters’ button.

### Exploring terminal exons of interest

Once specific filters have been applied by the user (as described above), the resulting set of terminal exons can be investigated in detail using different feature panels:

*‘Overview’ panel*: Presents a summary of the number of terminal exons found across tissues and cell types (Figure 2B).

*‘Metrics’ panel*: Provides the user with sample- and terminal exon-associated quality metrics as inferred by the STAR aligner (16) and TECtool (10), respectively (Figure 2B).

*‘Terminal Exons’ panel*: Offers an interactive table that contains detailed information about each terminal exon at single cell level (Figure 2C). The user can investigate and further filter terminal exons based on user-tailored features/columns, such as cell type, tissue, Ensembl/Entrez ID, etc. By default, the ‘Show only unique’ box is checked to prevent the user from looking at the same terminal exon identified within a multitude of single cells. The ‘UCSC terminal exon’ and ‘UCSC gene’ columns provide the option to investigate terminal exons and associated transcript isoforms in the UCSC Genome Browser (24). Finally, scTEA-db offers users to export their tailored views and filterings in the form of TSV or Excel files.

### General information and bulk data download

The ‘Info’ panel contains general information about scTEA-db and also provides an option for highly welcome user feedback. Finally, the full database in TSV file format and annotations in GTF are available as bulk data downloads (Figure 2B).

## Discussion

The great demand for transcript 3′ end annotations is reflected by the large number of resources that provide genomic locations and poly(A) site processing quantifications, many of which are heavily used by the community. Examples are PolyA_DB 3 (25), PolyASite 2.0 (18) and scAPAdb (26). Importantly, large numbers of the experimentally observed transcript 3′ ends are not covered by the current gene annotation and there exists no detailed information about the large number of not yet annotated terminal exon isoforms. However, the annotation of these isoforms is required to enable studying their expression in various systems of interest and to uncover their biological roles. Here, we have made use of a large number of publicly available full-length scRNA-seq datasets to create scTEA-db, a resource that provides the community with so far not yet annotated terminal exons and associated transcript isoforms at single cell, i.e. cell type resolution. scTEA-db is an easy to use web portal that offers user-specific searches, data views and downloads, thereby providing the foundation to study the biology of thousands of so far not yet annotated terminal exon isoforms. Our extended terminal exon annotation enables to infer the expression of the corresponding isoforms within any system of interest and thus also has the potential to contribute to more fine-grained cellular subtyping. Importantly, we have designed scTEA-db as an easy to expand resource that we aim to update by incorporating single cell sequencing datasets that will be made available by the community in the future. Additionally, providing expression information of the terminal exon transcript isoforms and corresponding protein domain predictions (27) will be valuable extensions. Finally, making use of bioinformatics tools for the prediction of RBP (28) and microRNA binding sites (29) within the 3′ UTRs of the terminal exons will further our understanding of the expression regulation and biological roles of specific isoforms in cell identity and function.

## Supplementary Material

gkad878_Supplemental_Files

## Data Availability

The data underlying this article are available in the article and in its online supplementary material. scTEA-db and the corresponding bulk data downloads are accessible at www.scTEA-db.org. The provided data were derived from datasets in the public domain. All dataset accession numbers are listed in Supplementary Tables S1 and S2. Single sample level accession number lists, the analysis source code and the source code for the scTEA-db web portal itself are available on Figshare (doi: https://www.doi.org/10.6084/m9.figshare.23946714).

## References

[1] Reyes A. , Huber W. Alternative start and termination sites of transcription drive most transcript isoform differences across human tissues. Nucleic Acids Res. 2018; 46:582–592. 29202200 10.1093/nar/gkx1165 PMC5778607

[2] Gruber A.J. , Zavolan M. Alternative cleavage and polyadenylation in health and disease. Nat. Rev. Genet. 2019; 20:599–614. 31267064 10.1038/s41576-019-0145-z

[3] Proudfoot N.J. , Brownlee G.G. 3′ non-coding region sequences in eukaryotic messenger RNA. Nature. 1976; 263:211–214. 822353 10.1038/263211a0

[4] Tian B. , Hu J., Zhang H., Lutz C.S. A large-scale analysis of mRNA polyadenylation of human and mouse genes. Nucleic Acids Res. 2005; 33:201–212. 15647503 10.1093/nar/gki158 PMC546146

[5] Berkovits B.D. , Mayr C. Alternative 3′ UTRs act as scaffolds to regulate membrane protein localization. Nature. 2015; 522:363–367. 25896326 10.1038/nature14321 PMC4697748

[6] Mayr C. Evolution and biological roles of alternative 3′ UTRs. Trends Cell Biol. 2016; 26:227–237. 26597575 10.1016/j.tcb.2015.10.012 PMC4955613

[7] Gruber A.J. , Schmidt R., Ghosh S., Martin G., Gruber A.R., van Nimwegen E., Zavolan M. Discovery of physiological and cancer-related regulators of 3′ UTR processing with KAPAC. Genome Biol. 2018; 19:44. 29592812 10.1186/s13059-018-1415-3 PMC5875010

[8] Taliaferro J.M. , Vidaki M., Oliveira R., Olson S., Zhan L., Saxena T., Wang E.T., Graveley B.R., Gertler F.B., Swanson M.S. et al. Distal alternative last exons localize mRNAs to neural projections. Mol. Cell. 2016; 61:821–833. 26907613 10.1016/j.molcel.2016.01.020 PMC4798900

[9] Gruber A.J. , Schmidt R., Gruber A.R., Martin G., Ghosh S., Belmadani M., Keller W., Zavolan M. A comprehensive analysis of 3′ end sequencing data sets reveals novel polyadenylation signals and the repressive role of heterogeneous ribonucleoprotein C on cleavage and polyadenylation. Genome Res. 2016; 26:1145–1159. 27382025 10.1101/gr.202432.115 PMC4971764

[10] Gruber A.J. , Gypas F., Riba A., Schmidt R., Zavolan M. Terminal exon characterization with TECtool reveals an abundance of cell-specific isoforms. Nat. Methods. 2018; 15:832–836. 30202060 10.1038/s41592-018-0114-z PMC7611301

[11] Picelli S. , Faridani O.R., Björklund A.K., Winberg G., Sagasser S., Sandberg R. Full-length RNA-seq from single cells using Smart-seq2. Nat. Protoc. 2014; 9:171–181. 24385147 10.1038/nprot.2014.006

[12] Leinonen R. , Sugawara H., Shumway M. The Sequence Read Archive. Nucleic Acids Res. 2011; 39:D19. 21062823 10.1093/nar/gkq1019 PMC3013647

[13] Kanz C. , Aldebert P., Althorpe N., Baker W., Baldwin A., Bates K., Browne P., van den Broek A., Castro M., Cochrane G. et al. The EMBL Nucleotide Sequence Database. Nucleic Acids Res. 2005; 33:D29–D33. 15608199 10.1093/nar/gki098 PMC540052

[14] Mölder F. , Jablonski K.P., Letcher B., Hall M.B., Tomkins-Tinch C.H., Sochat V., Forster J., Lee S., Twardziok S.O., Kanitz A. et al. Sustainable data analysis with Snakemake. F1000Research. 2021; 10:33. 34035898 10.12688/f1000research.29032.1 PMC8114187

[15] Cunningham F. , Allen J.E., Allen J., Alvarez-Jarreta J., Amode M.R., Armean I.M., Austine-Orimoloye O., Azov A.G., Barnes I., Bennett R. et al. Ensembl 2022. Nucleic Acids Res. 2021; 50:D988–D995. 10.1093/nar/gkab1049 PMC8728283 34791404

[16] Dobin A. , Davis C.A., Schlesinger F., Drenkow J., Zaleski C., Jha S., Batut P., Chaisson M., Gingeras T.R. STAR: ultrafast universal RNA-seq aligner. Bioinformatics. 2013; 29:15–21. 23104886 10.1093/bioinformatics/bts635 PMC3530905

[17] Wang L. , Wang S., Li W. RSeQC: quality control of RNA-seq experiments. Bioinformatics. 2012; 28:2184–2185. 22743226 10.1093/bioinformatics/bts356

[18] Herrmann C.J. , Schmidt R., Kanitz A., Artimo P., Gruber A.J., Zavolan M. PolyASite 2.0: a consolidated atlas of polyadenylation sites from 3′ end sequencing. Nucleic Acids Res. 2020; 48:D174–D179. 31617559 10.1093/nar/gkz918 PMC7145510

[19] Katz Y. , Wang E.T., Silterra J., Schwartz S., Wong B., Thorvaldsdóttir H., Robinson J.T., Mesirov J.P., Airoldi E.M., Burge C.B. Quantitative visualization of alternative exon expression from RNA-seq data. Bioinformatics. 2015; 31:2400–2402. 25617416 10.1093/bioinformatics/btv034 PMC4542614

[20] Pertea M. , Pertea G.M., Antonescu C.M., Chang T.-C., Mendell J.T., Salzberg S.L. StringTie enables improved reconstruction of a transcriptome from RNA-seq reads. Nat. Biotechnol. 2015; 33:290–295. 25690850 10.1038/nbt.3122 PMC4643835

[21] Chang W. , Cheng J., Allaire J., Sievert C., Schloerke B., Xie Y., Allen J., McPherson J., Dipert A., Borges B. et al. shiny: web application framework for R. 2023; https://github.com/rstudio/shiny.

[22] Wickham H. , Averick M., Bryan J., Chang W., McGowan L.D., François R., Grolemund G., Hayes A., Henry L., Hester J. et al. Welcome to the tidyverse. J. Open Source Softw. 2019; 4:1686.

[23] Maag J.L.V. gganatogram: an R package for modular visualisation of anatograms and tissues based on ggplot2. F1000Research. 2018; 7:1576. 30467523 10.12688/f1000research.16409.1 PMC6208569

[24] Meyer L.R. , Zweig A.S., Hinrichs A.S., Karolchik D., Kuhn R.M., Wong M., Sloan C.A., Rosenbloom K.R., Roe G., Rhead B. et al. The UCSC Genome Browser database: extensions and updates 2013. Nucleic Acids Res. 2013; 41:D64–D69. 23155063 10.1093/nar/gks1048 PMC3531082

[25] Wang R. , Nambiar R., Zheng D., Tian B. PolyA_DB 3 catalogs cleavage and polyadenylation sites identified by deep sequencing in multiple genomes. Nucleic Acids Res. 2018; 46:D315–D319. 29069441 10.1093/nar/gkx1000 PMC5753232

[26] Zhu S. , Lian Q., Ye W., Qin W., Wu Z., Ji G., Wu X. scAPAdb: a comprehensive database of alternative polyadenylation at single-cell resolution. Nucleic Acids Res. 2022; 50:D365–D370. 34508354 10.1093/nar/gkab795 PMC8728153

[27] Paysan-Lafosse T. , Blum M., Chuguransky S., Grego T., Pinto B.L., Salazar G.A., Bileschi M.L., Bork P., Bridge A., Colwell L. et al. InterPro in 2022. Nucleic Acids Res. 2023; 51:D418–D427. 36350672 10.1093/nar/gkac993 PMC9825450

[28] Lal A. , Galvao Ferrarini M., Gruber A.J. Investigating the human host–ssRNA virus interaction landscape using the SMEAGOL toolbox. Viruses. 2022; 14:1436. 35891416 10.3390/v14071436 PMC9317827

[29] McGeary S.E. , Lin K.S., Shi C.Y., Pham T.M., Bisaria N., Kelley G.M., Bartel D.P. The biochemical basis of microRNA targeting efficacy. Science. 2019; 366:eaav1741. 31806698 10.1126/science.aav1741 PMC7051167

