# Supplementary material for: scTEA-db: a comprehensive database of novel terminal exon isoforms identified from human single cell transcriptomes
Source: Nucleic Acids Res. 2023 Oct 18;52(D1):D1018–23. doi: 10.1093/nar/gkad878 (PMC10767918; doi:10.1093/nar/gkad878)
Supplement: gkad878_Supplemental_Files [file gkad878_supplemental_files.zip › Supplementary_Materials.pdf]

Supplementary Materials

Miguel Barquin[1], Ian U. Kouzel[1], Beat Ehrmann[1], Michael Basler[1,2], and Andreas J. Gruber[1,*]

[1] Department of Biology, University of Konstanz, 78464 Konstanz, Germany

[2] Biotechnology Institute Thurgau (BITg) at the University of Konstanz, Kreuzlingen, Switzerland

* To whom correspondence should be addressed. Tel: 0049 7531 880; Fax: 0049 7531 88 3688; Email: gruber[-at-]uni-konstanz[-dot-]de

Supplementary Figures

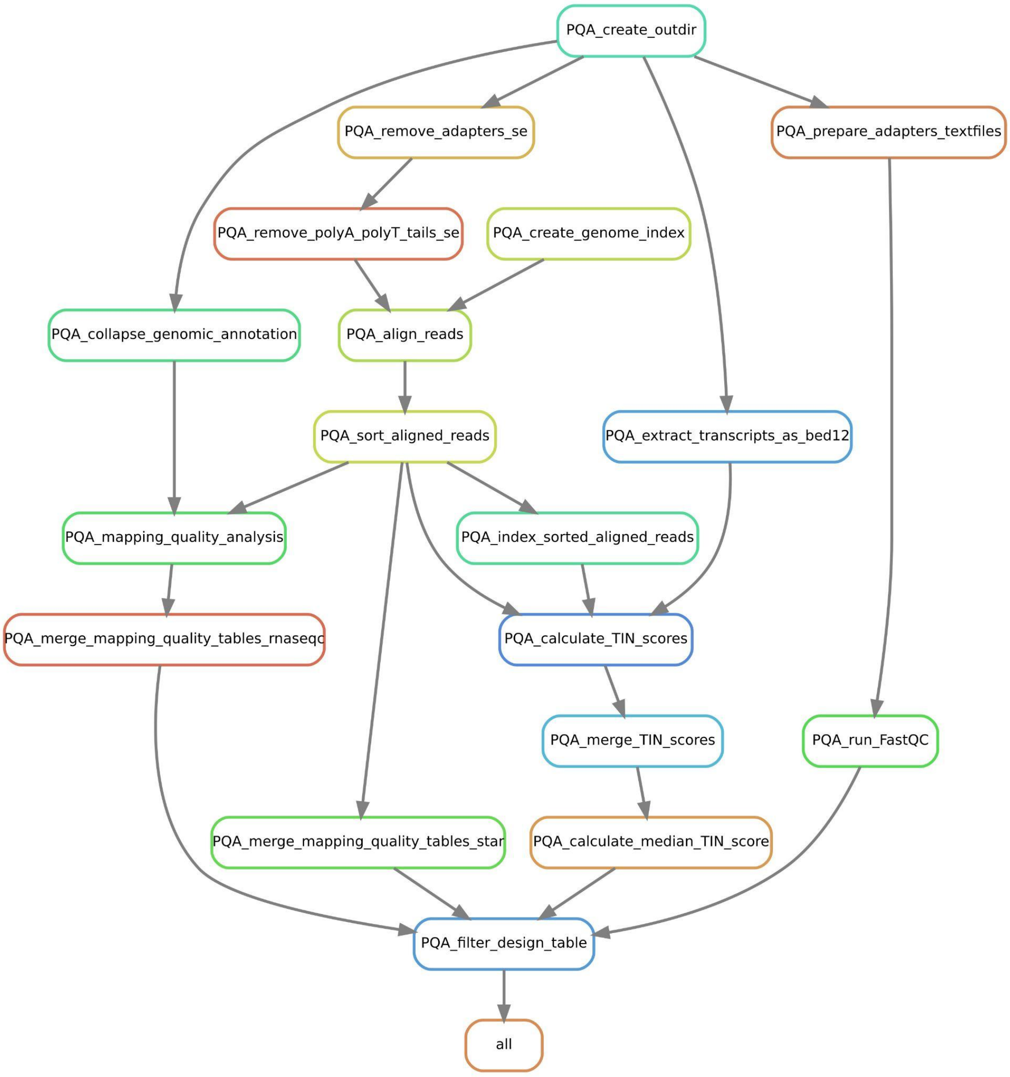

Supplementary Figure S1. Graphical representation of the computational Snakemake pipeline used for data quality control and filtering.

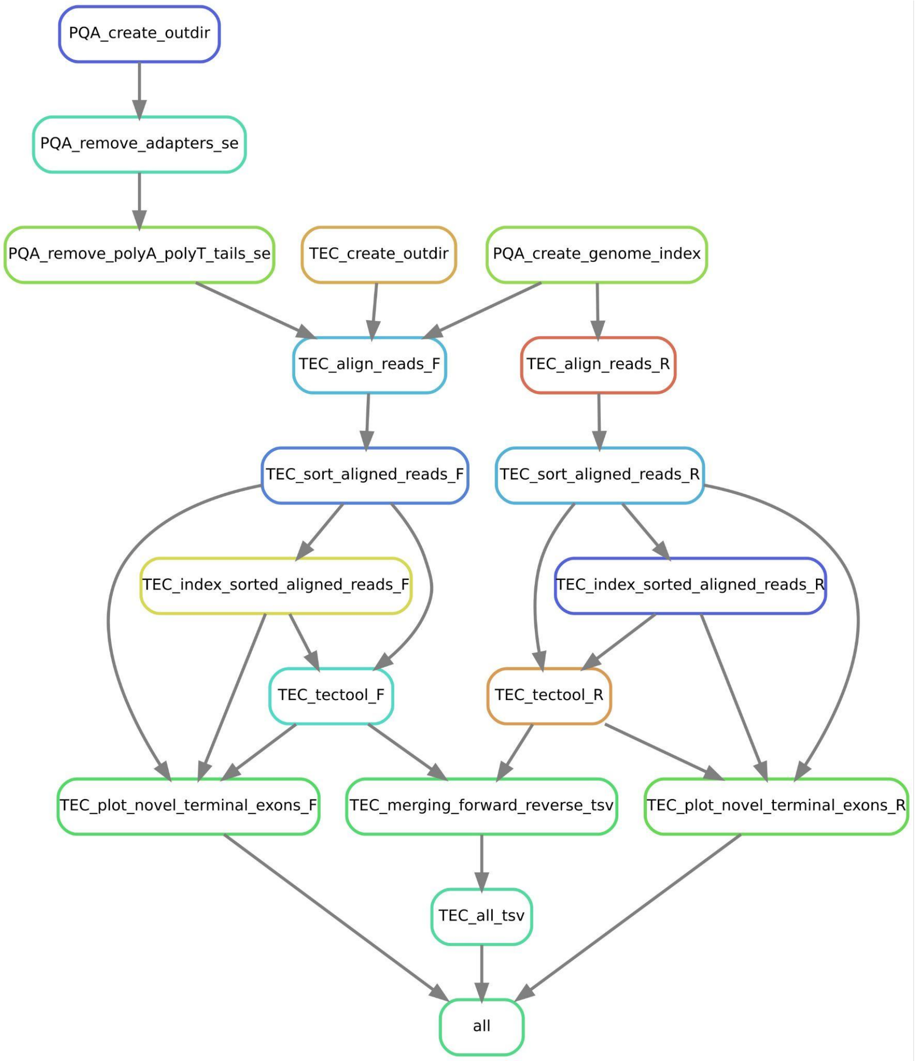

Supplementary Figure S2. Graphical representation of the computational Snakemake pipeline used to identify novel terminal exons.

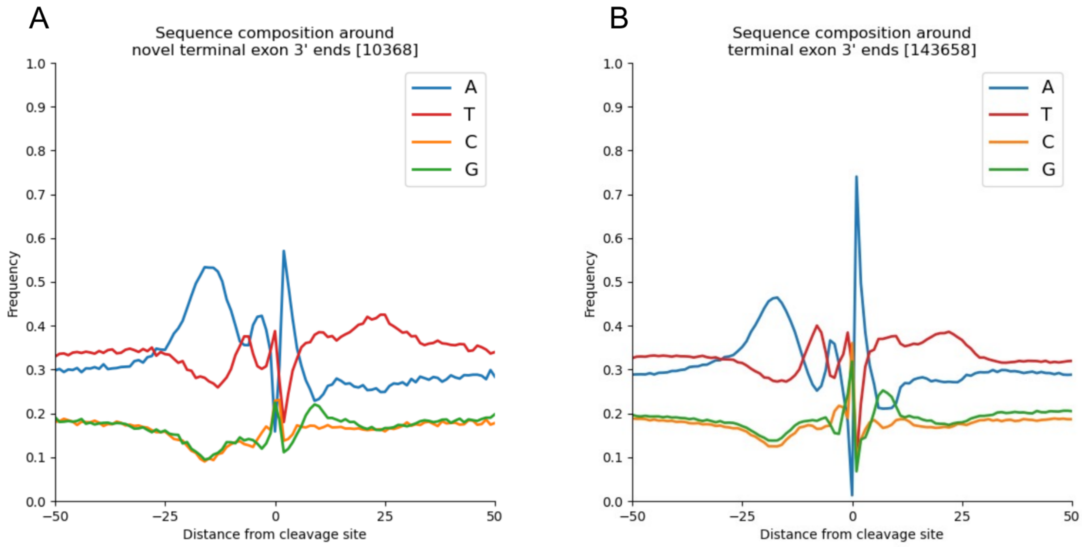

Supplementary Figure S3. Frequencies of nucleotides (nt) in the vicinity (+/- 50 nt) of **(A)** novel terminal exon 3' ends, and **(B)** 3' ends from the PolyASite 2.0 atlas (1) intersecting with previously annotated terminal exons. The number of 3' ends considered in (A) or (B), respectively, are indicated in squared brackets.

Supplementary Methods

Dataset selection criteria

Similar to 3' end sequencing protocols, also 3' tag-based single cell RNA sequencing (scRNA-seq) protocols sequence only the 3' end of transcripts. That is, while these protocols provide sequencing read coverage at the 3' end of terminal exons, they broadly lack sequencing read coverage at their 5' ends. Consequently, the 5' ends of terminal exons and the upstream exons they are spliced to can not be reconstructed from the sequencing reads obtained from 3' tag-based scRNA-seq protocols. In contrast, full-length transcript scRNA-seq protocols contain sequencing reads within the entire transcript body allowing to reconstruct terminal exons, including their exact 5' end and the upstream exon they are connected, i.e. spliced to. The currently most widely used full-length transcript scRNA-seq protocol is Smart-seq2 (2). Thus, for scTEA-db datasets available within the public domain were selected if they contained human Smart-seq2 datasets, whereas cells from complex disease were excluded (cancers, chronic viral infections, etc). That is, only datasets that included healthy

libraries or cells that do not belong to the above mentioned diseases were selected to be considered in our study. All details about the considered datasets, including accession numbers, tissue types, and cell types are listed in Supplementary Tables S1 and S2. Table 1 provides an overview of the number of considered datasets and cells per tissue.

Merging of GTF files obtained from individual single cells

To generate the final comprehensive scTEA-db GTF files the TECtool (3) GTF file entries for novel terminal exon 3' end isoforms obtained for individual cells were merged by making use of the StringTie tool version 2.2.1 (4). In addition to the resulting GTF file that contained all novel terminal exon 3' end isoforms ('scTEA_db_release_1_0.gtf') also a gene annotation enriched by these isoforms ('Homo_sapiens.GRCh38.102_scTEA_db_release_1_0.gtf') was created by merging the comprehensive GTF file with the Ensembl gene annotation version GRCh38.102 (5). Finally, in order to make the GTF files compatible with the UCSC genome browser (6) format specifications the term 'chr' was added as prefix to every chromosome and only entries for chromosomes chr1-22, X and Y were kept.

Analysis of nucleotide frequencies in the vicinity of poly(A) sites

Data collection: The GRCh38 release 110 unmasked genome assembly was obtained from Ensembl in FASTA file format (5). Human cleavage and polyadenylation (poly(A)) site coordinates annotated to be associated with terminal exons were obtained from the PolyASite 2.0 database (1) in BED file format. Novel terminal exon coordinates were extracted from the scTEA-db data bulk download file (www.scTEA-db.org; scTEA_db_release_1_0_20230809.tsv).

Poly(A) sites of novel terminal exons: The poly(A) site coordinates were extracted from the novel terminal exon data (see above) followed by collapsing the obtained sites in order to obtain a set of unique 3' end processing sites. Subsequently, analysis windows were defined spanning -200 nucleotides (nt) upstream to 200 nt downstream relative to the poly(A) sites and the corresponding sequences were extracted in fasta format utilizing the GRCh38 release 110 genome making use of pybedtools (7).

Poly(A) sites of known terminal exons: The poly(A) sites provided by the PolyASite atlas version 2.0 were filtered for sites with terminal exon (TE) annotation. For the analysis the representative poly(A) site coordinate was used (as provided in column 4 of the BED file). Again analysis

windows were defined in a similar manner as above, spanning -200 nt upstream to 200 nt downstream relative to the poly(A) sites and the corresponding genomic sequences (GRCh38 release 110) were extracted in FASTA format again utilizing pybedtools (7).

Creating nucleotide frequency plots: We conducted nucleotide frequency analysis for both categories of poly(A) sites mentioned above. Specifically, we quantified the nucleotide frequencies in the vicinity of novel terminal exon-associated poly(A) sites and of poly(A) sites annotated by the PolyASite atlas version 2.0, whereas only the region from 50 nt upstream to 50 nt downstream was visualized (Supplementary Figure S3).

References

1. Herrmann,C.J., Schmidt,R., Kanitz,A., Artimo,P., Gruber,A.J. and Zavolan,M. (2019) PolyASite 2.0: a consolidated atlas of polyadenylation sites from 3′ end sequencing. *Nucleic Acids Res.*

2. Picelli,S., Faridani,O.R., Björklund,A.K., Winberg,G., Sagasser,S. and Sandberg,R. (2014) Full-length RNA-seq from single cells using Smart-seq2. *Nat. Protoc.*, **9**, 171–181.

3. Gruber,A.J., Gypas,F., Riba,A., Schmidt,R. and Zavolan,M. (2018) Terminal exon characterization with TECtool reveals an abundance of cell-specific isoforms. *Nat. Methods*, **15**, 832–836.

4. Pertea,M., Pertea,G.M., Antonescu,C.M., Chang,T.-C., Mendell,J.T. and Salzberg,S.L. (2015) StringTie enables improved reconstruction of a transcriptome from RNA-seq reads. *Nat. Biotechnol.*, **33**, 290–295.

5. Cunningham,F., Allen,J.E., Allen,J., Alvarez-Jarreta,J., Amode,M.R., Armean,I.M., Austine-Orimoloye,O., Azov,A.G., Barnes,I., Bennett,R., *et al.* (2022) Ensembl 2022. *Nucleic Acids Res.*, **50**, D988–D995.

6. Meyer,L.R., Zweig,A.S., Hinrichs,A.S., Karolchik,D., Kuhn,R.M., Wong,M., Sloan,C.A., Rosenbloom,K.R., Roe,G., Rhead,B., *et al.* (2013) The UCSC Genome Browser database: extensions and updates 2013. *Nucleic Acids Res.*, **41**, D64–9.

7. Dale,R.K., Pedersen,B.S. and Quinlan,A.R. (2011) Pybedtools: a flexible Python library for manipulating genomic datasets and annotations. *Bioinformatics*, **27**, 3423–3424.
